# Supplementary figures and images for: Isolation and sequence-based characterization of a koala symbiont: Lonepinella koalarum
Source: PeerJ. 2020 Oct 20;8:e10177. doi: 10.7717/peerj.10177 (PMC7583611; doi:10.7717/peerj.10177)

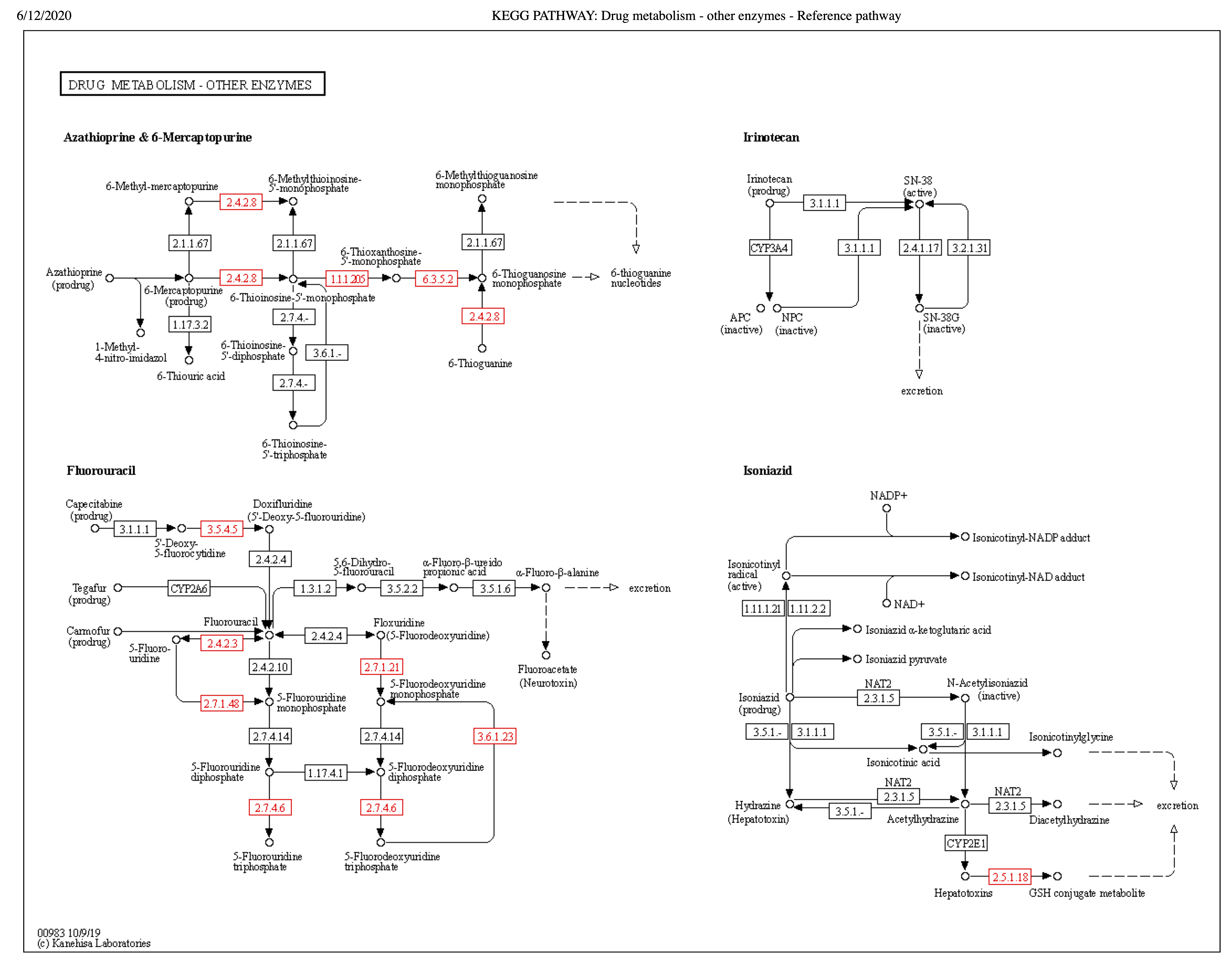

Supplement: Supplemental Information 1 — Putative genes in this metabolism were mapped onto the reference pathway map ko00983 using the KEGG webtool. Enzymes in red show positive hits in the assembly of L. koalarum strain UCD-LQP1. Kanehisa Laboratories, 2017. Drug metabolism - Reference pathway. Kyoto Encyclopedia of Genes and Genomes. Available at https://www.genome.jp/kegg-bin/show_pathway?map=map00983&show_description=show (accessed 12 June 2020). [file peerj-08-10177-s001.jpg]

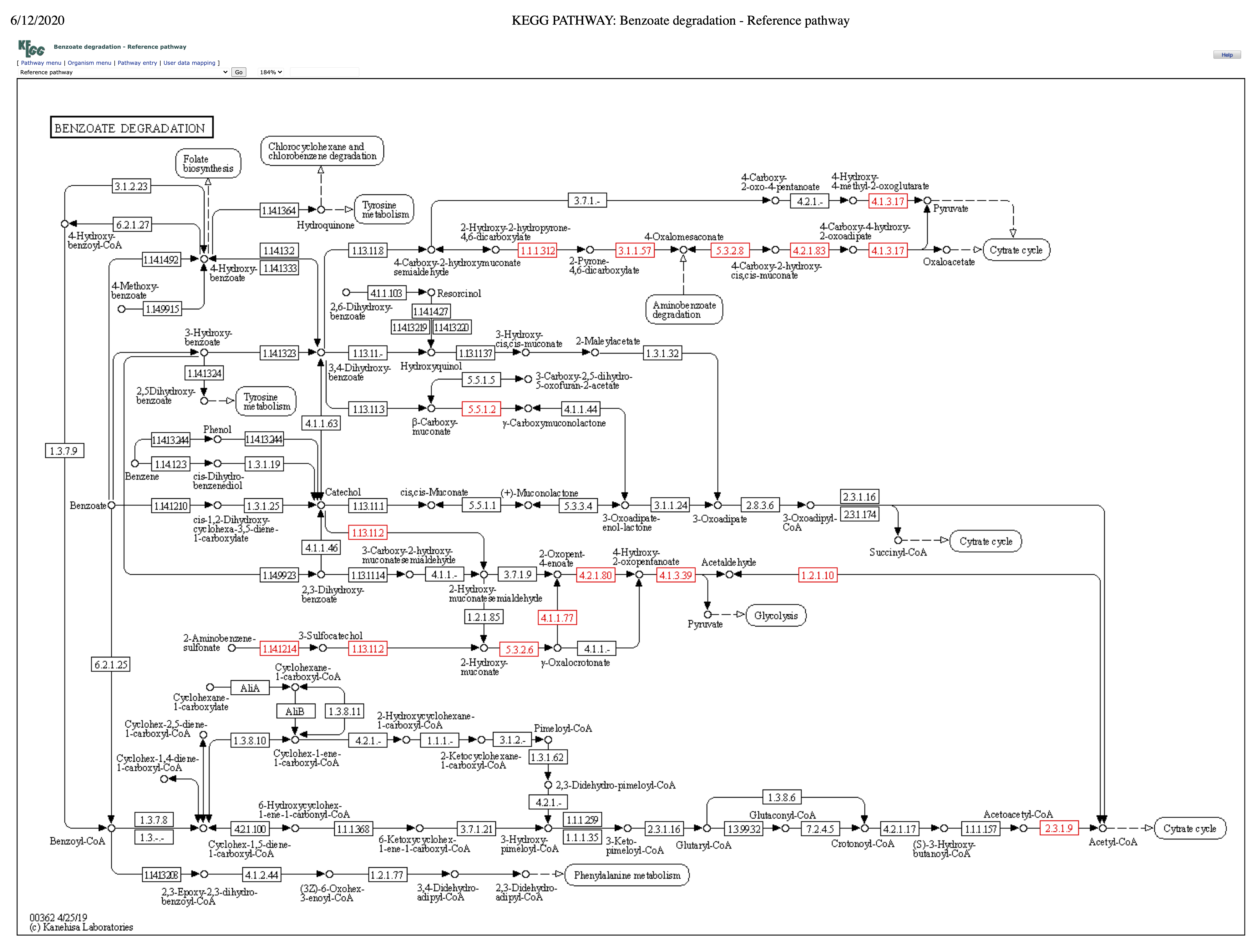

Supplement: Supplemental Information 2 — Putative genes in this metabolism were mapped onto the reference pathway map ko00362 using the KEGG webtool. Enzymes in red show positive hits in the assembly of L. koalarum strain UCD-LQP1. Kanehisa Laboratories, 2017. Benzoate degradation - Reference pathway. Kyoto Encyclopedia of Genes and Genomes. Available at https://www.genome.jp/kegg-bin/show_pathway?map00362 (accessed 12 June 2020). [file peerj-08-10177-s002.jpg]

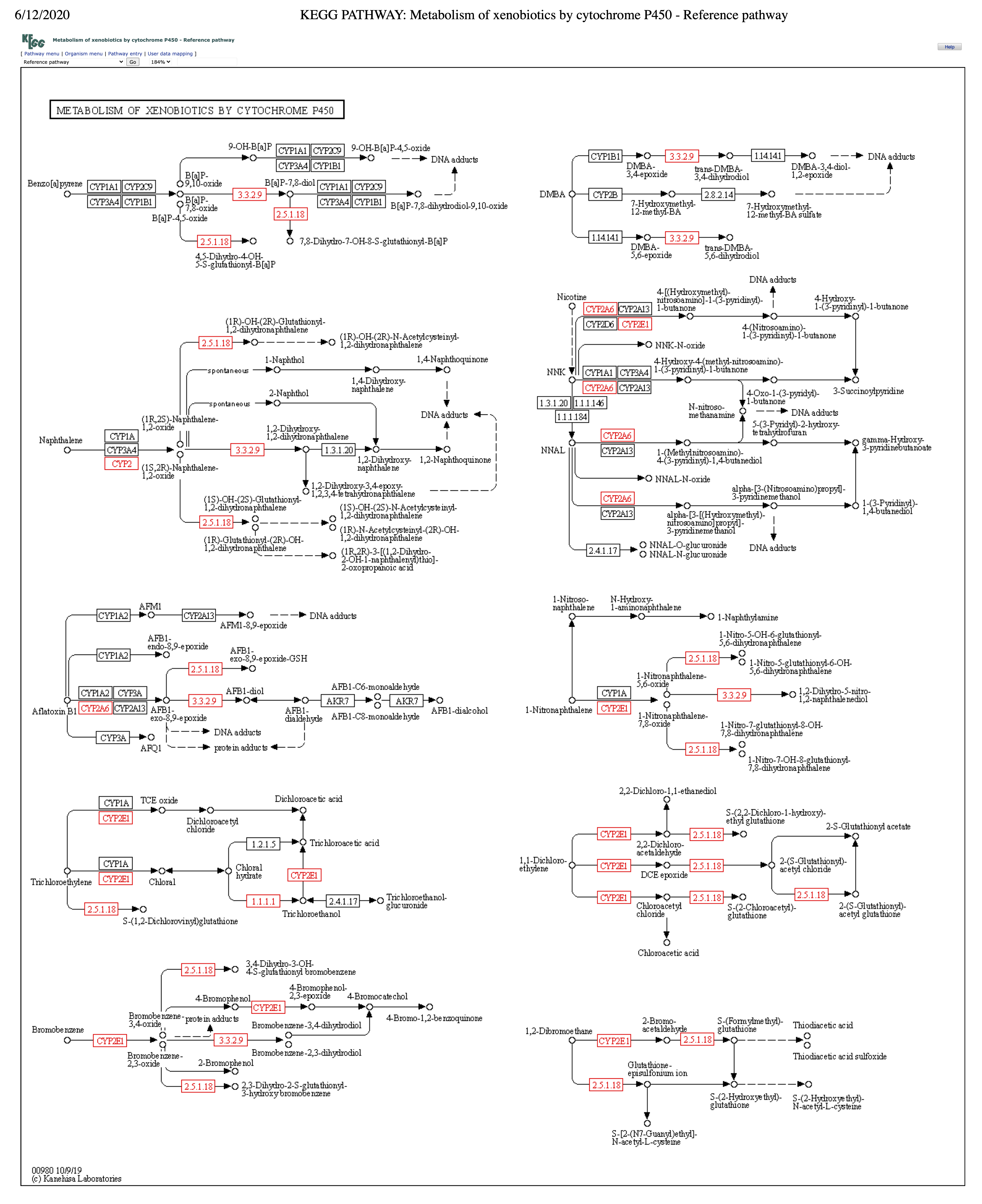

Supplement: Supplemental Information 3 — Putative genes in this metabolism were mapped onto the reference pathway map ko00980 using the KEGG webtool. Enzymes in red show positive hits in the assembly of L. koalarum strain UCD-LQP1. Kanehisa Laboratories, 2017. Metabolism of xenobiotics by cytochrome P450 - Reference pathway. Kyoto Encyclopedia of Genes and Genomes. Available at https://www.genome.jp/kegg-bin/show_pathway?map00980 (accessed 12 June 2020). [file peerj-08-10177-s003.jpg]

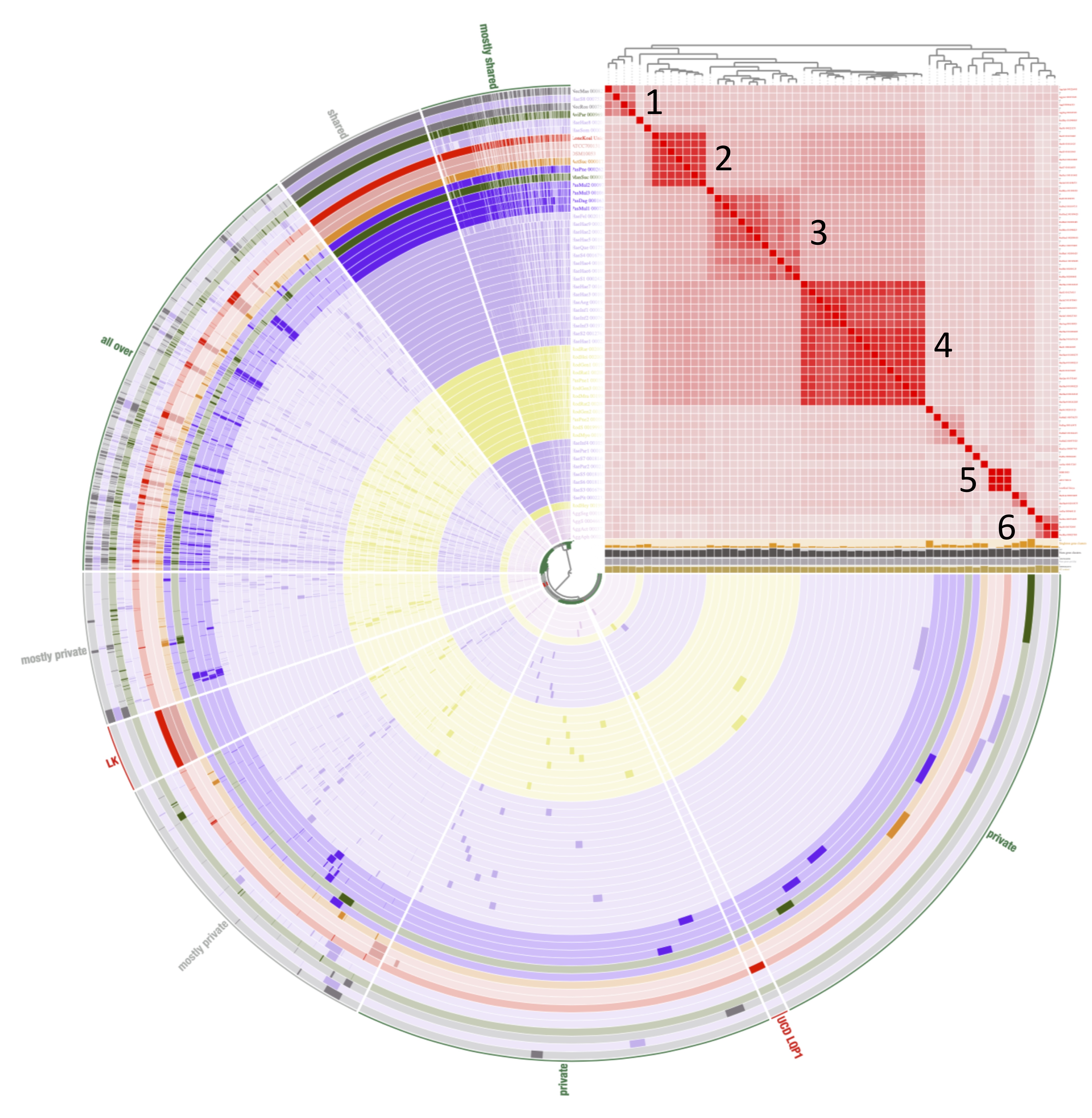

Supplement: Supplemental Information 4 — This figure shows the same as Fig. 2 in the main manuscript with the only difference that ANI values in the heatmap were colored in red when > 70% instead of > 95%. Numbers correspond to clusters of genomes discussed in the main manuscript. [file peerj-08-10177-s004.jpg]
